# How should healthcare be reported in Catalonia? Qualitative study with healthcare leaders

**DOI:** 10.1186/s12913-022-08718-4

**Published:** 2022-11-23

**Authors:** Anna García-Altés, Hortensia Aguado, Mercedes Guilabert, Irene Carrillo, Jose Joaquín Mira

**Affiliations:** 1 Agència de Qualitat i Avaluació Sanitàries de Catalunya (AQuAS) ES, Barcelona, Spain; 2 Universidad Miguel Hernández de Elche ES, Elche, Spain; 3 Atenea Research Group, Foundation for the Promotion of Health and Biomedical Research, Sant Joan d´Alacant, Spain

**Keywords:** Reporting, Benchmarking, Focus groups, Qualitative research, Healthcare quality

## Abstract

**Background:**

The Results Centre is the name of a project that, since 2012, has been openly publishing the results of each healthcare centre in Catalonia, with the idea of promoting benchmarking among centres and transparency toward society. As the project evolves, it has become increasingly necessary to adapt its contents and formats. The objective of this study is to identify the preferences and expectations of healthcare leaders regarding the Results Centre.

**Methods:**

A qualitative study was conducted using the nominal group technique. Five nominal groups were created with the participation of 58 professionals (26 from hospital care, 16 from primary care, and 16 from long-term care centres). The areas of analysis were: (1) what the Results Centre of the future should be like; (2) what information needs should be addressed; and (3) what novelties should be incorporated to stimulate quality improvement. The spontaneity of ideas, intensity of recommendations, and intergroup consistency were analysed. The study was conducted in April 2019.

**Results:**

The requirements reported by the participants to be met by the Results Centre included: being a tool for benchmarking and strategic decision-making; adjusted and segmented indicators; non-clinical information (patient experience, socio-economic status, etc.); and data accessible to all stakeholders, including citizens. The ideas were consistent across the different levels of care, although the intensity of recommendations varied depending on their content.

**Conclusions:**

Regional agencies that are accountable for health outcomes should be consistently committed to adapting to the needs of different stakeholders in the health system. This project is an example of how this requirement has been addressed in Catalonia.

## Introduction

It has been shown that comparison between healthcare centres contributes to an improvement in results and economic savings by improving the adequacy of the procedures. These savings could be used to redirect efforts toward the needs of the population with accessible and affordable services [1–4]. A driver for quality improvement is reputation [5]. The key issue is to produce reliable information on quality robust in the face of criticism from providers and understandable and available to the public [6]. This information, presented in a summarised form, focusing on the key indicators, in a clear and accessible format, can be a determining factor in hospital choice for citizens [7, 8].

One of the most famous benchmarking exercises was the publication of the Coronary Artery Bypass Grafting (CABG) mortality results by hospitals and surgeons in New York State in 1989, which demonstrated a positive impact on results [9]. The pioneering effort in Europe took place in Scotland in 1994, with the publication of the annual reports of clinical outcomes, although with a much lower impact [10].

In Catalonia, since 2012, the results of each healthcare centre, (primary care centres, hospitals, long-term care centres, etc.) according to a broad set of indicators, have been published and are open to healthcare professionals and citizens to promote benchmarking among centres and transparency with society. This project, called the “Central de Resultats” (Results Centre), offers feedback on the performance of the centres on a nominal basis, based on relevant measures to enhance the quality of care. Furthermore, it identifies the trends, emerging problems, and experiences of successful healthcare practice and management [11]. It includes more than 60 indicators for each type of centre across different aspects of healthcare, such as patient satisfaction, effectiveness, appropriateness, efficiency, IT use, etc. The project is led by the Catalan Agency for Health Quality and Evaluation (AQuAS), a publicly funded entity independent from the Catalan Health Department.

As the project has evolved, it has become increasingly necessary to adapt the information held in the Results Centre, regarding the technical and communicative aspects related to professionals and citizenship, respectively. In 2015, to identify its strengths and weaknesses, as well as to determine the usefulness of the project, a survey aimed at hospital managers was conducted. The results showed that 97.4% of the respondents knew of the project and it was used in the governing bodies of hospitals (91.4%) and quality improvement commissions (77.1%) [12]. A year later, in 2016, various qualitative studies were conducted to identify information needs regarding the Results Centre. These included three focus groups held with citizens (26 people), semi-structured interviews with 16 managers, healthcare professionals, communication professionals, and citizens, and a consensus dynamic with 33 opinion leaders. Among other issues, citizens identified their reference health professional as their interlocutor, also regarding related to health information and data regarding the quality of the healthcare system [13]. At that time, we collaborated with citizens to design the infographic of the project that presented the main yearly results of the healthcare system [14].

It is essential for the project to reach and meet the needs of professionals. Thus, in 2019 we sought to go a step further and fill this gap. Hence, this study was conducted to identify the preferences and expectations of healthcare leaders in primary care, hospital care, and long-term care centres regarding the contents and format of the Results Centre.

## Methods

### Design

A qualitative study was conducted based on the content and discourse analysis of healthcare professionals and leaders or managers of the three levels of care in the Catalan healthcare system (primary, hospital, and long-term care). The study was conducted using the nominal group technique [15, 16] since it aimed to achieve individual productivity of the ideas, combined with an interactive voting system that allowed a hierarchical structuring of the ideas. The study was conducted from 9 to 10 April, 2019.

### Scope and participants

#### Research group

The promoter and research group consisted of six professionals linked to health (five women and one man). The professional profile was one health economist, one nurse, and four psychologists (three doctors). Of the six researchers, two belonged to the institution that coordinated the Results Centre project (AQuAS), and the others from an external institution with experience in qualitative research. Of the latter four, MG and JJM were responsible for conducting the qualitative techniques with support from the other two external professionals. The project promoters (AGA and HA) attended the nominal groups only as observers and did not intervene, except for logistical matters. None of the facilitators of the qualitative technique knew the participants beforehand. No contractual ties mediated the relationship between any members of the promoter group and the study participants.

#### Nominal groups

The inclusion criteria used (healthcare leaders) was related to the representativeness, professional profile, interest, and knowledge of the Results Centre. The participants were selected to ensure the representativeness of the three healthcare settings (primary, hospital, and long-term care) and the different types of providers and territories. AQuAS personnel were responsible for the participants’ selection and invitation to participate. An e-mail that detailed the study and the place and time of the meeting was sent and confirmation of attendance was requested. All the professionals voluntarily agreed to participate, for which no financial remuneration was provided.

Eighty-two health professionals (34 from primary care, 32 from hospital care, and 16 from long-term care) were invited to participate. Finally, 58 professionals attended the nominal groups (response rate 70.7%). There were 24 technicians (41.4%), 22 clinicians (37,9%), and 12 managers (20.7%). Of these, 36 were women (62.1%) and many were aged 45 years or older (Table 1). The distribution of the participants by nominal groups was as follows: 16 primary care representatives in two groups of eight (response rate 61.5%), 26 hospital care representatives in two groups of 13 (response rate 81.3%), and 16 long-term care representatives in one group (response rate 100%).

**Table 1:** Description of the participants in the nominal groups

		Primary care	Hospital care	Long-term care	Total
*Sex*	*Age*				
Woman	18-44 y	3	7	5	15
	≥ 45 y	7	8	6	21
Man	18-44 y	1	4	2	7
	≥ 45 y	5	7	3	15
*Professional category*					
Technician		6	8	10	24
Clinician		9	9	4	22
Manager		1	9	2	12

### Procedure

The research team held the nominal groups at the AQuAS facilities in perfectly equipped rooms. The group sessions focused on the different aspects of the information in the Results Centre, the functions that the project should fulfil, and those that it does not currently fulfil (Table 2). Each group session lasted approximately 150 minutes and followed a question script. The questions were determined by consensus between the research group and AQuAS.

**Table 2:** Question script for each level of care and consistency of the common ideas

Questions	Level of Care	Consistency
How do you imagine/would you like the Results Centre to be in 5 years?	Primary care, hospital care, long-term care	Future Results Centre
Do you consider the areas/themes of the Results Centre to be useful? Should they be maintained or changed?	Hospital care	Information needs
What information needs do primary care centres have?	Primary care	Information needs
What information needs will the long-term care centres have in the medium term?	Long-term care	Information needs
What functions and services (utilities) should the Results Centre have in long-term care setting?	Long-term care	How to stimulate quality improvement
What novelties should the Results Centre incorporate to stimulate quality improvement in the centres?	Primary care, hospital care, long-term care	How to stimulate quality improvement

The sessions began with a presentation by the AQuAS leaders who provided a description of the study and the objectives intended to be achieved at that meeting. Next, the participants were asked to provide consent to their responses being audio recorded to facilitate the subsequent analysis. Once they accepted and signed the informed consent form, they introduced themselves. This was followed by the group discussion.

The moderator asked the first question from the script to begin the discussion. Participants then engaged in a period of individual reflection for approximately 10 minutes, during which they wrote their ideas and proposals on the cards. They returned the cards to the moderator, who shuffled them before reading them aloud to maintain the anonymity. As the cards were put together, the moderator placed them on a panel visible to all participants. During this phase, the moderator asked the participant who formulated an idea for clarification. Similar ideas were discussed among the experts who proposed them to determine whether they referred to the same idea or were nuances.

The last phase consisted of prioritisation of the ideas proposed in the previous phase (related only to questions 2 and 3). A computerised voting system was used, and the participants individually scored each idea on a scale of 1 (I do not agree at all with the proposed idea) to 5 (I very much agree with the proposed idea). After the vote, the system provided a visualisation of the results to identify the ideas that the group considered most important. This process was repeated for two questions. A summary of the results obtained was sent to all the participants to could correct any misunderstandings or errors.

### Data analysis

Information obtained in each category was classified according to the frequency of the comments (how often the same idea was independently repeated) and consistency (whether the same idea was repeated in different groups). For questions 2 and 3, using the prioritisation system, the following values were indicated for each idea: frequency, intensity of the recommendation (scoring assigned to each idea by the experts) and agreement regarding the recommendation (coefficient of variation). The ideas were coded and categorised by two researchers (MG and IC). Any discrepancies or disagreements were resolved by a third researcher.

Once the study was completed, the participants received a report of the results for potential future discussions.

Although numerical data were presented, they corresponded to a nominal scale and should be interpreted as considering the order rather than the magnitude of the data.

## Results

### What should the future Results Centre look like?

The ideas that the experts spontaneously and independently reported most frequently were being a tool for knowledge exchange (benchmarking) and continuous improvement (*n* = 42), which reflected the complexity of the system and health determinants (adjusted and disaggregated data) (*n* = 39), incorporated the vision of citizens (*n* = 36), and facilitated strategic decision-making (*n* = 30). Other ideas that appeared consistently across the three levels of care were related to desirable qualities (transparency and reliability), types of content (new indicators and data adjustments), issues related to information format and support, and dissemination of information to stakeholders (Table 3).

**Table 3:** Ideas on what the future Results Centre should look like

Idea	Frequency			
	Total	PC	HC	LTC
Tool for knowledge exchange (benchmarking) and continuous improvement	42	12	16	14
Reflection of system complexity and health determinants (adjusted indicators); comprehensive and disaggregated view of the system	39	6	15	18
Incorporate citizens’ vision; open and adapted to the citizens	36	19	13	4
Facilitating tool for decision making linked to strategy (planning and management) and action	30	8	20	2
Trustworthy, reliable	27	3	13	11
Interactive, customisable, and user-friendly information and platform; useful and actionable data and graphics	26	14	10	2
New indicators	26	4	6	16
Territorial information (small and large scale) with graphical presentation	21	11	8	2
Periodicity	18	5	10	3
Unification of sources and criteria in data recording and calculation of indicators; alignment of different systems and entities	18	9	7	2
Indicators that reflected processes and continuity of care	17	3	8	6
Visibility and dissemination of results at all levels (social, scientific, and international)	12	3	5	4
Information useful for professionals	7	3	2	2

Frequency: Number of times the same idea was repeated independently

*PC* Primary care, *HC* Hospital care, *LTC* Long-term care.

### What information should be addressed by the Results Centre?

At the three levels of care (primary, hospital, and long-term), main information needs were the incorporation of non-healthcare information (socio-economic determinants of health, human resources, efficiency of the system, patient-reported experience measures –PREMs- and patient-reported outcome measures -PROMS-, quality of life, etc.) (*n* = 21), contextualisation of indicators based on standardised parameters to enable comparison between centres (*n* = 14), and orientation towards key processes (*n* = 6). However, the sensitivity of the groups to these needs was different. The addition of non-care information was most demanded by primary and long-term care professionals, while process orientation was mainly recognised in hospital care. Other needs were related to the interactivity of and access to the system, availability of the measures of the effectiveness of interventions, selection of key indicators by consensus, improvement of the quality of the records, and adjustment of data by territory (Table 4).

**Table 4:** Ideas on what information needs should be addressed by the Results Centre

Idea	Frequency				Intensity and agreement Mean (CV)		
	Total	PC	HC	LTC	PC	HC	LTC
Incorporation of non-healthcare information (socio-economic determinants of health, human resources, efficiency of the system, PROM, PREM, quality of life, comfort, etc.)	21	9	1	11	4.4 (0.1)	2.3 (0.7)	4.2 (0.2)
Adjustment and contextualisation of the indicators to allow comparison between centres; elaboration of tailor-made reports with standardised parameters according to different areas.	14	4	6	4	3.9 (0.2)	4.4 (0.2)	4.5 (0.2)
Interactive and dynamic system that allowed continuous monitoring and improved the periodicity, format, and channel of information presentation	8	3	5	–	4.4 (0.2)	4.5 (0.2)	–
Mainstreaming and the need to communicate to the public	7	3	4	–	4.5 (0.1)	3.4 (0.4)	–
Segmented information oriented to key processes	6	1	4	1	2.8 (0.5)	4.9 (0.1)	2.3 (0.5)
Unification and homogeneity of reliable sources; integration of indicators	6	4	2	–	3.8 (0.2)	4.1 (0.2)	–
Resolution and cross-source linked data to enable decision-making based on feedback on the effectiveness of interventions	6	4	2	–	4.5 (0.2)	3.5 (0.4)	–
Redesign of the system so it is not a repository of indicators; review and refinement of some indicators according to the ‘less is more’ principle	3	1	2	–	4.0 (0.2)	4.0 (0.3)	–
Need to validate the Minimum Basic Data Set and other sources of information	2	–	1	1	–	4.4 (0.1)	3.2 (0.5)
Territory-adjusted indicators	2	1	1	–	3.6 (0.2)	4.2 (0.2)	–
Inclusion of desirable and relevant indicators as judged by those who interpret the data	2	1	1	–	4.1 (0.1)	3.6 (0.3)	–
Easy and differentiated access to the scorecard of indicators for managers, professionals, and the public	2	1	1	–	4.0 (0.1)	2.5 (0.5)	–

Frequency: Number of times the same idea was repeated independently

Intensity: Mean of the scores assigned to each idea by the experts.

Agreement: Coefficient of variation.

*CV* Coefficient of variation, *PC* Primary care, *HC* Hospital care, *LTC* Long-term care.

### What novelties should be incorporated into the Results Centre to stimulate quality improvement?

The innovations consistently identified in all three settings were the adoption of a benchmarking approach (*n* = 12), more frequent updating of information (*n* = 11), incorporation of person-centred care indicators (*n* = 7), adjustment of data according to patient profile (*n* = 6), and provision of valuable outcomes for practitioners (n = 6). However, there were other ideas that appeared consistently across two levels of care were more frequently reported. Among them, hospital care professionals requested new channels and formats for information (*n* = 15), long-term care professionals emphasised the desire for information grouped by territory and area of influence (*n* = 12), and primary care professionals identified a need for greater social dissemination of data from the Results Centre among the population and professionals (*n* = 11). Other proposals mentioned information that empowered action, standardised the construction of indicators, and improved the alignment of information with objectives (Table 5).

**Table 5:** Ideas on what novelties should be incorporated into the Results Centre to stimulate quality improvement

Idea	Frequency				Intensity and agreement Mean (CV)		
	Total	PC	HC	LTC	PC	HC	LTC
New channels and formats for message dissemination	18	3	15	–	4.1 (3)	4 (0.2)	–
Information grouped at the territorial level and according to each area of influence	14	–	2	12	–	4.4 (0.2)	4.6 (0.1)
Greater social dissemination among the population and professionals	12	11	1	–	4.0 (0.2)	3.9 (0.3)	–
Reports focused on continuous improvement and benchmarking	12	4	5	3	4.8 (0.1)	4.5 (0.1)	4.7 (0.1)
Regular and more up-to-date information	11	3	4	4	4.0 (0.3)	4.3 (0.2)	4.0 (0.2)
Indicators of health outcomes and person-centred care (quality of life, PREM, PROM, etc.)	7	1	5	1	3.9 (0.2)	4.0 (0.2)	2.6 (0.5)
Contextualised information according to patient profile for comparison purposes	6	1	3	2	4.7 (0.1)	4.0 (0.2)	3.1 (0.4)
Results that add value and were shared by the professionals involved	6	2	1	3	4.1 (0.1)	4.2 (0.2)	4.0 (0.3)
Actionable information for the management of practitioners	5	4	1	–	3.7 (0,3)	3.2 (0,4)	–
Standardisation of the construction of indicators	5	4	1	–	4.0 (0,2)	4.2 (0.2)	–
Information aligned with the objectives	2	–	1	1	–	3.9 (0.3)	2.1 (0.3)

Frequency: Number of times the same idea was repeated independently

Intensity: Mean of the scores assigned to each idea by the experts.

Agreement: Coefficient of variation.

*CV* Coefficient of variation, *PC* Primary care, *HC* Hospital care, *LTC* Long-term care.

## Discussion

### Statement of the principal findings

This study aimed to identify the preferences and expectations of healthcare leaders in primary, hospital, and long-term care centres regarding the contents and format of the Results Centre. The main views of professionals were that it should be an instrument for benchmarking and continuous improvement, which should incorporate non-care information (socio-economic determinants of health, human resources, system efficiency, PROM, PREM, quality of life, comfort, etc.) and needed new channels and formats for the diffusion of messages. Furthermore, it should be a tool for decision-making, which allowed clinical management and planning in centres, adjusted to the reality of the healthcare system, and aligned with the Department of Health’s policies. The Results Centre should make it possible to identify areas for improvement, orientation for change, and assessment of political priorities.

### Interpretation within the context of wider literature

Measurement of the quality in healthcare is a complex issue [17]. No single indicator will adequately summarise the quality of an organisation. Hence, improving the way in which healthcare performance is measured and information is used is central to healthcare improvement [18].

Once an objective, reliable, sensitive, and valid system of indicators is obtained, further challenges arise, such as to identify the most efficient channels of dissemination, the different subgroups of target audiences, specify their information needs, and verify that the information shared achieved its intended purpose: contribution to quality improvement. Briefly, to obtain optimal results, it is necessary to answer two questions. First, how to select and compare indicators comprehensible and useful for target audiences, which involves determining what information is relevant and of interest, how to facilitate understanding, and what purpose the results will have. Second, ways to disseminate this information through the most efficient channel [19].

The Results Centre should provide an image of the healthcare system that reflects its high level of complexity. The information must be segmented by context, patient profile, and social determinants of health. Moreover, public disclosure should be a tool to support quality initiatives in place [20] and measure and benchmark professional groups’ quality of care and contribution in achieving populations’ health improvement and healthcare system sustainability, such as nurses through nursing-sensitive indicators [21, 22].

The professionals who participated in this study wanted the Results Centre to include important results for the public (such as PREMs and PROMs), assess the different providers, and be accountable for how public resources are used. In reality, limited existing benchmarking efforts in Europe include indicators built with user feedback. Quality dimensions, other than safety and effectiveness, are systematically under-represented, and there is poor coverage of many clinical specialities or indicators not related to hospital care [23]. Knowing patients’ opinions would enhance strategies designed to improve the quality of service provision [24]. Citizens participation in decision-making is an increasing reality in healthcare systems [25].

Despite efforts to make healthcare performance information understandable to the professionals and public, there is still room for improvement. Professionals who participated in this study asked for an interactive easy-to-use web with online information. Presenting benchmarking information easily accessible for users can enhance the role of quality of care and provider decisions [26]. For professionals and managers, it is an additional push to compare themselves with their peers to encourage quality improvement [27]. The use of data by more professionals, patients, and institutions should be an important goal to foster improvement [28, 29]. In the public sector, having access to administration data fosters transparency, efficiency, and equal opportunities while creating value [30].

### Strengths and limitations

A strength of this study was the use of nominal group techniques to obtain the opinions of leading healthcare professionals. This technique combined the advantages of individual work with those of group work and avoided the pressures that a group can exert on the individuals, which made it possible for participants to freely express their opinions [31]. The moderator sought to minimise bias by encouraging participation and free expression of ideas at each session. A relevant feature of this technique was that the subjects did not interact directly and the engagement of the group was limited to specific issues, such as the clarification of individual proposals to others, the generation of new ideas, and their organisation [32].

Another strength was the participation of leading professionals from the Catalan healthcare sector, who represented the diversity of centres, territories, types of organisation, and employers. These professionals expressed their opinions free of group pressure.

This study has some limitations. First, those inherent to qualitative research. The participants who participated in the study could be the most sensitized to the topic addressed. Some answers could have been affected by the pressure of group work. The representation of the different care settings in the existing indicators for the time of the study was uneven. This aspect could have affected the motivation of the groups (e.g., all long-term care professionals invited agreed to participate). In some cases, the vision provided by the professionals could be individualistic and focused only on the aspects applicable or that affected their setting. Second, qualitative research is an exploratory approach to the elements that need to change based on the views of the lead participants; however, this is not a substitute for data or other information. Although, the high degree of consensus achieved among the wide range of healthcare leaders who participated minimised this issue. Moreover, we ensured our reporting adhered to the Consolidated Criteria for Reporting Qualitative Studies (COREQ) guidelines.

A practical limitation, although related to the context than to the study itself, was the difficulty in implementing some proposals, such as those directly related to the availability of data (i.e., higher frequency of reports). For the Results Centre to truly function as a tool for self-assessment and continuous improvement, the frequency of information updating must be higher (either monthly or quarterly). This is the highest priority in the development of the project.

### Implications for policy, practice, and research

The Results Centre measures and disseminates the results achieved in healthcare by different providers to facilitate responsible decision-making in the provision of quality healthcare in Catalonia. In addition to being a reference tool in the evaluation of public policies, it is an instrument for the continuous improvement of all agents to achieve sustainable public health and excellence. This is a pioneering initiative in Spain [33]. Similar projects have long been in place in the United Kingdom, the Netherlands, Germany, Australia, the United States, and Canada, among others. Thus, the Results Centre is aligned with the most advanced countries regarding transparency and accountability in healthcare [34, 35].

A year ago, the proposals identified were prioritised for introduction in the project and priority was given to improve the presentation of results, in indicators and the periodicity of information. Unfortunately, the emergence of the COVID-19 pandemic had a significant impact on the project and halted all new developments. However, all new actions are planned to restart soon.

This qualitative study revealed a line of work within the Results Centre, which incorporated the opinions and needs of the main agents involved.

The future project involves prioritising these opinions and needs and transforming and revising the current indicators and drawing up the Future Central Results Centre.

## Conclusions

The participation of the stakeholders (such as citizens and professionals) proved to be a key element in the Results Centre [31, 32]. The Results Centre, based on the firm conviction of the need for and goodness of transparency and accountability, must be constantly evolving. It should seek to improve the selection and accessibility of those indicators that will allow citizens to better approach the complex reality of the care process and health outcomes. In particular, the participation of professionals in project design by means of qualitative dynamics, such as in this study, is a success. It is an excellent way to push forward the improvement of the project based on the viewpoint of high-profile users. The common goal of improving day-to-day work will stimulate, to an even greater extent, health professionals, managers, and governing bodies of healthcare centres to continue seeking opportunities for improvement.

## Data Availability

This is a qualitative study. All data generated or analysed are included in this published article.

## Abbreviations

CV: Coefficient of variation
PC: Primary care
HC: Hospital care
LTC: Long-term care
